# A microarray analysis of retinal transcripts that are controlled by image contrast in mice

**Published:** 2007-06-18

**Authors:** Christine Brand, Frank Schaeffel, Marita Pauline Feldkaemper

**Affiliations:** Section for Neurobiology of the Eye, University Eye Hospital Tuebingen, Tuebingen, Germany

## Abstract

**Purpose:**

The development of myopia is controlled by still largely unknown retinal signals. The aim of this study was to investigate the changes in retinal mRNA expression after different periods of visual deprivation in mice, while controlling for retinal illuminance.

**Methods:**

Each group consisted of three male C57BL/6 mice. Treatment periods were 30 min, 4 h, and 6+6 h. High spatial frequencies were filtered from the retinal image by frosted diffusers over one eye while the fellow eyes were covered by clear neutral density (ND) filters that exhibited similar light attenuating properties (0.1 log units) as the diffusers. For the final 30 min of the respective treatment period mice were individually placed in a clear Perspex cylinder that was positioned in the center of a rotating (60 degrees) large drum. The inside of the drum was covered with a 0.1 cyc/degree vertical square wave grating. This visual environment was chosen to standardize illuminances and contrasts seen by the mice. Labeled cRNA was prepared and hybridized to Affymetrix GeneChip® Mouse Genome 430 2.0 arrays. Alterations in mRNA expression levels of candidate genes with potential biological relevance were confirmed by semi-quantitative real-time reverse transcription polymerase chain reaction (RT-PCR).

**Results:**

In all groups, *Egr-1* mRNA expression was reduced in diffuser-treated eyes. Furthermore, the degradation of the spatial frequency spectrum also changed the *cFos* mRNA level, with reduced expression after 4 h of diffuser treatment. Other interesting candidates were *Akt2*, which was up-regulated after 30 min of deprivation and *Mapk8ip3*, a neuron specific JNK binding and scaffolding protein that was temporally regulated in the diffuser-treated eyes only.

**Conclusions:**

The microarray analysis demonstrated a pattern of differential transcriptional changes, even though differences in the retinal images were restricted to spatial features. The candidate genes may provide further insight into the biochemical short-term changes following retinal image degradation in mice. Because deprivation of spatial vision leads to increased eye growth and myopia in both animals and humans, it is believed some of the identified genes play a role in myopia development.

## Introduction

Myopia is increasing in prevalence world-wide [1], but information remains fragmented about the regulation of ocular growth and the development of refractive errors. In animal models, it has been shown that the alteration of retinal images leads to altered gene expression and a change in eye growth patterns. Myopia can be artificially induced by placing negative lenses in front of the eye or by reducing the retinal image quality by a diffuser [2,3], whereas hyperopia can be generated by positive lens wear [4]. These eye growth responses were shown in several animal models, including chicks [2], tree shrews [5], marmosets [6] and rhesus monkeys [7]. In mice, a shift toward myopia can be induced by form deprivation [8-10]. During negative spectacle lens wear, axial eye growth rates are accelerated until a sharp retinal image is achieved. Depriving the retina of high contrast and high spatial frequencies by diffusers also induces axial eye growth, but a sharp retinal image cannot be restored in this case. A local mechanism within the retina is involved in eye growth regulation since neither accommodation nor contributions from the brain are necessary [11].

Little is known about genes and proteins whose expression is susceptible to altered visual stimulation. Among the known genes and substances are glucagon [12,13], retinoic acid [14], vasointestinal peptide (Vip) [15-17], sonic hedgehog (Shh) [18,19], and the transcription factor Egr-1 [20,21]. The identification of novel retinal genes that are influenced by altered visual conditions is important as they may lead to new targets for a pharmacological therapy of myopia. In the present study, DNA-microarrays, followed by semi-quantitative real-time RT-PCR, were used to screen for differentially regulated genes and subsequent validation.

## Methods

### Animals

Black wildtype C57BL/6 male mice were raised on a 12 h: 12 h light-dark cycle with light onset at 8:00 a.m. The mice were reared in the local animal facility and given free access to water and food. For the GeneChip® experiments the mice (3 per group) were studied at postnatal (P) days 30 and 32, while the mice used for the validation of the results by semi-quantitative real-time RT-PCR (6 animals per group) were studied at P29 to P36. The experimental treatment was in accordance with the ARVO Statement for the Use of Animals in Ophthalmic and Vision Research and was approved by the university commission for animal welfare (reference AK 3/05).

### Diffuser and neutral density filter design

Diffusers were made from transparencies that were sanded down with emery paper to give a frosted appearance. Neutral density (ND) filters of 0.1 log unit light attenuation (Kodak Wratten Gelatin Filter No. 96; Rochester, NY) were used to match image illumination [21,22]. Velcro rings with an inner diameter of 6 mm and a total diameter of 10 mm were fitted with the frosted transparency or ND filter. The contralateral eyes served as controls to minimize effects due to differences between individual animals in factors such as hormone level and immunological state.

### Treatment procedure

**Figure 1:**
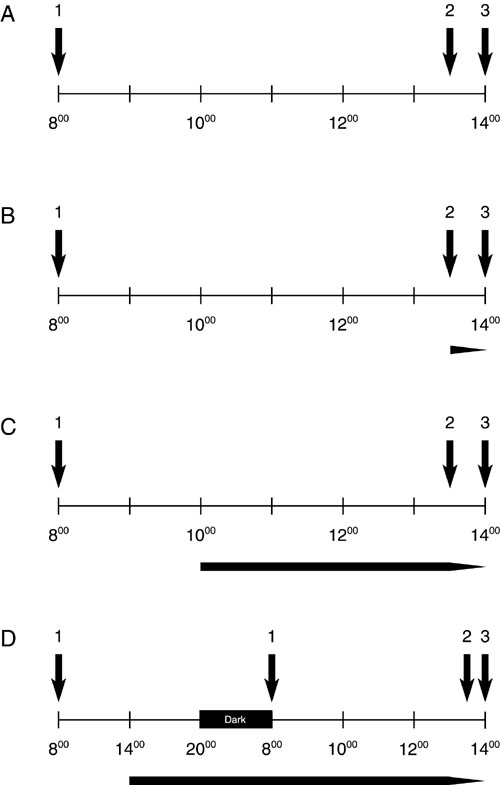
Detailed representation of the treatment protocol. **A**: shows the protocol for the control animals. **A**-**D**: An arrow above each time bar signals (1) the time when mice were brought into light, (2) when each mouse was put into the rotating drum, and (3) when mice were sacrificed. **B**-**D**: The arrows below the time bars mark the beginning of each treatment: 30 min (**B**), 4 h (**C**), and 6+6 h (**D**) diffuser and ND filter attachment. Three animals were treated per day, and were brought into light as follows: the first mouse at 8.00 a.m., the second at 8.30 a.m., and the third animal at 9.00 a.m. Accordingly, the entire treatment schedule was shifted 30 min or 1 h later (to the right) for the 2nd and 3rd animals, respectively.

Velcro rings were attached to the periorbital fur the day before the experiments while animals were under diethyl ether anesthesia. For anesthesia, animals were put in a diethyl ether containing glass jar supplied with a grid at the bottom. The glass jar was covered by a lid until the animals were unconscious (which takes about 20-30 s). The complementary Velcro rings carrying the diffusers and ND filters were attached on the experimental day. Mice were kept under cool white light of approximately 120 lux (Lumilux 30W/840; Osram, Munich, Germany) during the treatment period and were sacrificed after a total of six hours in light (Figure 1). At the end of the respective treatment period, each mouse was separately placed on a stationary platform in the center of the rotating drum with a diameter of 60 cm for 30 min. Illumination in the drum was about 400 lux. The stripe pattern had a spatial frequency of 0.1 cycles per degree. The angular velocity was about 60 degrees per second, which was much higher than that used by Prusky et al. [23]. Previous studies in our lab, using the drum, have shown that an angular velocity of 50 to 60 deg/s was optimal for studying spatial vision in C57BL/6 mice in our optomotor set-up [24].

The whole body optomotor grating acuity of C57BL/6 mice was recently measured in the same drum and was found to be limited to approximately 0.3-0.4 cycles/degree. The stripe contrast measured at 400 lux was approximately 90% [24]. Previous experiments have shown that the image contrast with the diffusers used in the current study is reduced to about 60-70% of the initial value [25]. The illumination before and during the experiment was far below the illumination that was previously used to induce retinal degeneration in mice [26]. To insure that gene expression changes were a result of the treatment and not the hormone status, age, light, or time of day, we only used male litter mates and sacrificed the mice after they had been exposed to 6 h of light. This was usually between 2 and 3 p.m. in the afternoon (Figure 1).

### Tissue preparation and RNA isolation

All mice were sacrificed by an overdose of diethyl ether. Their eyes were enucleated and immediately transferred to a petri dish containing chilled Ringer's solution until preparation. The eyes were subsequently placed on a filter paper, perforated by a canula and opened with scissors, cutting around the iris. The lens was removed, and the retina was extracted, snap frozen in liquid nitrogen, and stored at -70 °C. The preparation was done using a stereo boom microscope (10X magnification) and RPE cells were carefully removed. The total length of time between death/enucleation and tissue freezing was about 5-10 min. Each retina was treated and analyzed as a separate sample. The retinas were homogenized for 1 min with speed increasing from 11,000 to 20,000 rpm (Diax 900 Homogenizer; Heidolph, Kelheim, Germany). Total RNA was isolated with a kit (RNeasy Mini Kit; Qiagen, Hilden, Germany) according to the manufacturer's instructions. RNA quality and quantity were determined by the Agilent 2100 Bioanalyzer (Agilent Technologies, Palo Alto, CA), which yielded RNA integrity numbers (RIN) from 8.4 to 9.3.

### Microarray analysis

The GeneChip® mouse genome 430 2.0 Array from Affymetrix (Santa Clara, CA) allows the comprehensive analysis of genome-wide expression on a single array. We used 45,000 probe sets to analyze the expression level of over 39,000 transcripts and variants from over 34,000 well-characterized mouse genes.

Microarray analysis was performed by the Affymetrix Resource Facility at the University of Tuebingen as follows. Target labeling for the expression analysis was performed for 1 μg total RNA per sample according to the manufacturer's protocol (Affymetrix). The GeneChips® were automatically stained and washed in a fluidics station as recommended by the manufacturer. The scanning and the analysis were done using the Affymetrix Microarray Suite Software (v. 5.0).

### Affymetrix data analysis and statistics

The relative abundance of individual genes is based on the signal intensities of the corresponding probe sets that were analyzed by ArrayAssist 4.0 (Stratagene, La Jolla, CA). The microarray data were not Benjamini/Hochberg corrected since no significant differences in gene expression were obtained when this procedure was performed. Normalization and confirmation is covered in the Discussion section. Data from the experiments involving 30 min, 4 h and 6+6 h treatments were individually normalized using robust multi-array analysis with correction for GC content (gcRMA). The strategy of this normalization method is the calculation of a background adjustment step that ignores the mismatch (MM) intensities but incorporates sequence information from the probes (GC content) [27]. Afterwards the data were analyzed group-wise by paired t-tests to find genes that were differentially expressed in the diffuser- and filter-treated eyes of the same animal. The p-value was below 0.05 and a minimum fold-change (FC) was 1.5. mRNA level changes observed throughout the diffuser treatment were evaluated by normalizing together raw data from all three microarray experiments (30 min, 4 h, 6+6 h). One kind of background adjustment was calculated for all the 18 arrays. The quality of all microarray experiments was assessed by GAPDH 3'/5' and Actin 3'/5' ratios as well as background, scalar factors and present calls (Table 1) [28].

**Table 1:** Quality data of the GeneChip® experiments.

	**Array 30 min**	**Array 4 h**	**Array 6+6 h**
GAPDH 3'/5' ratio	0.74±0.03	0.89±0.01	0.79±0.02
Actin 3'/5' ratio	1.53±0.07	1.8±0.08	1.68±0.03
Background	51.13±6.59	80.52±6.94	81.68±18.5
Scalar factor	1.56±0.38	1.02±0.14	1.34±0.08
% present	56.69±1.34	54.45±1.19	52.4±1.44

The quality of each chip is evaluated and depends (among others) on 3'/5' ratios, the background and scalar factor. The percentage of present calls is also an important indicator of quality. Shown are the mean values of six arrays per treatment group with corresponding standard deviation (SD). All values met the expected quality criteria.

### Network generation and pathway analysis

The networks and functional analyses were generated through the use of Ingenuity Pathways Analysis (Ingenuity® Systems).

A data set containing gene identifiers and corresponding fold change values was uploaded into the application. Each gene identifier was mapped to its corresponding gene object in the Ingenuity Pathways Knowledge Base. These genes, called focus genes, were overlaid onto a global molecular network developed from information contained in the Ingenuity Pathways Knowledge Base. Networks of these focus genes were then algorithmically generated based on their connectivity.

### Semi-quantitative real-time polymerase chain reaction

All RNA samples used for semi-quantitative RT-PCR were treated with RNase-free DNase I (Roche, Mannheim, Germany). Gel electrophoresis was used to check quality of RNA. Concentration and purity were determined by spectrophotometry at 260 and 280 nm (average ratio 1.95±0.08). Of each sample, 1 μg total RNA was reverse transcribed (M-MLV reverse transcriptase; Promega, Madison, WI) using a combination of 50 ng random hexamers and 500 ng oligo (dT)_15_ primers in a total volume of 20 μl.

Gene sequences chosen for validation were obtained from the National Center for Biotechnology Information NCBI. Primers (Table 2) were preferentially designed to bind within the same coding region as the Affymetrix probes using a commercial program (Prime; GeniusNet Husar; KYE Systems, Heidelberg, Germany) and Primer Premier 5 (Premier Biosoft International; Palo Alto, CA). They were ordered from a commercial synthesis service (VBC Genomics, Vienna, Austria).

**Table 2:** Description of primers.

**Accession number**	**Gene**	**Sense primer (5'-3')**	**Antisense (5'-3')**	**Amplicon**
M12481	beta-actin	GGCTATGCTCTCCCTCACG	CTTCTCTTTGATGTCACGCACG	144 bp
X15267	Arp	CCTCCTTCTTCCAGGCTTTG	GGCTCCCACCTTGTCTCC	104 bp
AK009241	Krt2-6b	GCTCACATCACGATTCACACA	GACAGGAAGGTTTATGAGGTTG	220 bp
NM_007434	Akt2	GAGGACAATGACTATGGGCG	TTCAGCAGTCCAGCCAGCA	191 bp
NM_033597	Myb	CCAGGAGAAGCATTATTTTGA	AACCATAGCAGCGAACACAT	254 bp
NM_013931	Mapk8ip3	ACTCCATCCTCACCAGTCCT	AGAGAGAGCAAAGGGTTGGA	123 bp
M20157	Egr-1	TAGCAGCAGCAGCACCAGC	CATAGGGTTGTTCGCTCGGC	101 bp
NM_010234	Fos	CCCTGTGAGCAGTCAGAGAA	GGTGTGTTTCACGAACAGGT	145 bp
XM_126772	Usp36	GCAGGACCTAATTCAGCACA	TGGGTGCTTGCTCTCTTCAT	139 bp
XM_355521	Zmym4	TTCAGTCAGTGGCAGTCCTCT	ACAACCAGAGCAAGAAACTCG	130 bp
NM_172803	Dock4	GCTTACCTGGAAGGCAGTG	CATCCACAACTGTTTGCTTT	192 bp
NM_010638	Klf9	GCCCACTGTGTGAGAAGAGA	TGTCAGTCTGTTTCCTGGGA	192 bp
NM_172406	Trak2	GCTGAGATTGAAGGGACCAT	AGTGTCATTGGCAACCTTGA	114 bp
NM_019635	Stk3	ATCCCTACAAACCCACCAC	GGCTCTCTGCTCAGGACTCT	105 bp
NM_010200	Fgf13	TGAACAGCGAGGGATACTTG	TTCACATGGTTGCCTTTCAT	184 bp
NM_013822	Jagged1	AACACCCGAACTGGACAAAT	GCCCACTGTCTGCTATACGA	95 bp

Shown are Genbank accession numbers, sequences and amplicon lengths of the primers used.

PCR was performed in a thermocycler (iCycler; Bio-Rad, Hercules, CA) using a commercial fluorescence detection kit (QuantiTect SYBR Green PCR kit; Qiagen). After an initial heat activation (15 min at 95 °C) 40 cycles of 15 s at 94 °C, 30 s at 59 °C and 45 s at 72 °C were run. Cyclic fluorescence measurements were taken at the end of the annealing phase. The volume of a single reaction added up to 15 μl containing 2 ng template and a final primer concentration of 0.6 μM each. All PCR products were verified by automated sequencing or by restriction enzyme digestion (*Usp36*). Both the acidic ribosomal protein (*Arp*) and β-actin were chosen as internal reference genes and used for subsequent analyses.

### Semi-quantitative real-time reverse transcriptase polymerase chain reaction data analysis and statistics

Measurements were performed in triplicates and the C_T_ (Threshold Cycle) means were used for further data analyses by following a procedure described in reference [29].

Briefly, the efficiencies (E) of the individual primer sets during PCR were determined by dilution series and following equation: E=10^(-1/slope)^.

Statistical data analysis was performed after conversion of the raw data into normalized expression (NE). Therefore, the C_T_ means were transformed into relative quantities (RQ), and corresponding normalization factors (NF) were determined.

For the RQs, all C_T_ values obtained for a single gene were considered (e.g. control, 30 min, 4 h, and 6+6 h group). The sample showing the highest expression level (e.g. the lowest C_T_) was set to 1 (reference sample, RS). All other RQs were calculated by following formula:

Relative Quantity (Sample, S)=Efficiency^(CT (RS)-CT (S))^.

The normalization factor represents the geometric mean (root) of the reference gene RQs:

NF=(RQ(β-actin)xRQ(Arp))^1/2^

The NE represents the ratio of the relative quantity of a sample and its corresponding normalization factor: NE=^RQ^/_NF_

NE differences between diffuser-treated and ND filter-covered fellow eyes were calculated and statistically analyzed by un-paired Student's t-tests against null.

## Results

### GeneChip® results

GeneChip® analysis was performed on three animals per treatment group. mRNA expression levels in the diffuser-covered eyes were calculated relative to those in the ND filter-treated fellow eyes. After gcRMA normalization, expression of 16 genes was found to be affected after 30 min, with 13 genes being up-regulated. The 4 h treatment resulted in 27 differentially expressed genes, 23 of which were down-regulated. After 6+6 h, the number of up- and down regulated genes was balanced, with 10 genes being up-regulated and 11 being down-regulated.

The genes were arranged in a list according to their major function as determined by Ingenuity Pathways Analysis (Table 3).

**Table 3:** List of genes obtained by paired analysis and gcRMA normalization.

**Treatment 30 min**		
**Gene ID**	**FC**	**Gene title**
**Transcription regulator (3)**		
13653	-1.65	early growth response 1 (Egr-1)
22141	1.51	tubby homolog (mouse; Tub)
17260	1.63	MADS box transcription enhancer factor 2, polypeptide C (Mef2c)
**Transporter (3)**		
73836	1.53	solute carrier family 35, member B2 (Slc35b2)
18824	2.00	proteolipid protein 2 (colonic epithelium-enriched; Plp2)
224022	2.26	solute carrier family 7, member 4 (Slc7a4)
**Transmembrane receptor (1)**		
22288	1.59	utrophin (homologous to dystrophin; Utrn)
**Peptidase (1)**		
69617	-1.55	pitrilysin metallopeptidase 1 (Pitrm1)
**Enzyme (3)**		
545195	1.50	cytochrome P450, family 4, subfamily f, polypeptide 16 (Cyp4f16)
69719	1.70	carbamoyl-phosphate synthetase 2, aspartate transcarbamylase, and dihydroorotase (Cad)
54616	1.72	exostoses (multiple)-like 3 (Extl3)
**Other (4)**		
26936	-1.58	myosin phosphatase-Rho interacting protein (m-Rip)
57908	1.52	zinc finger protein 318 (Zfp318)
320165	1.52	transforming, acidic coiled-coil containing protein 1 (Tacc1)
320295	1.58	RIKEN cDNA C920006O11 gene (C920006O11Rik)
**Unknown (1)**		
1427797_s_at	1.62	
**Treatment 4 h**		
**Gene ID**	**FC**	**Gene title**
**Transcription regulator (5)**		
13653	-2.20	early growth response 1 (Egr1)
14281	-2.05	FBJ murine osteosarcoma viral oncogene homolog (cFos)
225872	-1.91	neuronal PAS domain protein 4 (Npas4)
21418	-1.52	transcription factor AP-2 alpha (Tcfap2a)
21677	1.56	TEA domain family member 2 (Tead2)
**Transporter (1)**		
317717	-1.59	SEC22 vesicle trafficking protein homolog A (S. cerevisiae; Sec22l2)
**Peptidase (1)**		
72344	1.61	ubiquitin specific peptidase 36 (Usp36)
**Kinase (1)**		
56637	-1.52	glycogen synthase kinase 3 beta (Gsk3b)
**Enzyme (2)**		
12662	-1.74	choroideremia (Rab escort protein 1; Chm)
78797	-1.52	NADPH dependent diflavin oxidoreductase 1 (Ndor1)
**Other (14)**		
19707	-2.27	RALBP1 associated Eps domain containing 1 (Reps1)
68607	-2.01	serine hydrolase-like 2 (Serhl)
67785	-1.95	zinc finger, MYM-type 4 (Zmym4)
22289	-1.71	ubiquitously transcribed tetratricopeptide repeat, X chromosome (Utx)
104910	-1.63	chromosome 14 open reading frame 68 (C14ORF68)
67974	-1.56	chromosome 10 open reading frame 9 (C10ORF9)
15982	-1.53	interferon-related developmental regulator 1 (Ifrd1)
70828	-1.53	RIKEN cDNA 4633401B06 gene (4633401B06Rik)
109200	-1.51	RIKEN cDNA A430102J17 gene (A430102J17Rik)
77656	-1.51	RIKEN cDNA C430045I18 gene (C430045I18Rik)
216971	-1.51	chromosome 17 open reading frame 63 (C17ORF63)
320003	-1.50	RIKEN cDNA C430014K11 gene (C430014K11Rik)
78893	1.53	CCR4-NOT transcription complex, subunit 10 (Cnot10)
52710	1.54	G protein-coupled receptor 172A (Gpr172b)
Unknown (3)		
1443057_at	-2.41	
1446932_at	-1.83	
1444274_at	-1.60	
**Treatment 6+6 h**		
**Gene ID**	**FC**	**Gene title**
**Transcription regulator (2)**		
13653	-1.77	early growth response 1 (Egr1)
109115	-1.67	suppressor of Ty 3 homolog (S. cerevisiae; Supt3h)
**Transporter (1)**		
224022	-1.55	solute carrier family 7, member 4 (Slc7a4)
**Enzyme (5)**		
74335	-1.90	X-ray repair complementing defective repair inChinese hamster cells 3 (Xrcc3)
50505	-1.69	excision repair cross-complementing rodent repair deficiency, complementation group 4 (Ercc4)
19361	-1.68	RAD51 homolog (RecA homolog, E. coli; S. cerevisiae; Rad51)
13804	-1.68	endonuclease G (Endog)
242202	1.64	phosphodiesterase 5A, cGMP-specific (Pde5a)
**Other (12)**		
627191	-1.87	transmembrane protein 90a (Tmem90a)
237781	-1.52	Smith-Magenis syndrome chromosome region, candidate 7 (Smcr7)
76073	-1.51	polycomb group ring finger 5 (Pcgf5)
19650	-1.51	retinoblastoma-like 1 (p107; Rbl1)
210766	1.55	BRCA1/BRCA2-containing complex, subunit 3 (Brcc2)
238130	1.56	dedicator of cytokinesis 4 (Dock4)
20324	1.58	serum deprivation response (phosphatidylserine binding protein; Sdpr)
320191	1.61	hook homolog 3 (Drosophila; Hook3)
67693	1.61	Huntingtin interacting protein K (Hypk)
19359	1.65	RAD23 homolog B (S. cerevisiae; Rad23b)
211550	1.69	TRAF-interacting protein with a forkhead-associated domain (Tifa)
73126	1.72	RIKEN cDNA 3110038A09 gene (3110038A09Rik)
**Unknown (1)**		
1440123_at	1.61	

"Entrez gene" accession numbers or Affymetrix ID (Gene ID), fold changes (FC) and names of the genes that were differentially expressed in the diffuser covered eyes relative to the ND filter treated control eyes are displayed. A p<0.05 was considered significant. Genes chosen for validation are colored in red.

To identify diffuser specific genes that were temporally regulated, the raw data of all three microarray experiments were normalized together and expression changes were determined by pair-wise comparisons. The p-value was set to 0.01 and a minimum fold change of 2.0 was established. The expression of 249 genes was significantly different after 6+6 h versus 30 min of treatment, and that of 191 genes was significantly different after 4 h versus 30 min of treatment but the expression of only 49 genes was significantly different after 6+6 h versus 4 h of treatment. Six genes were chosen to confirm the temporal expression analysis: fibroblast growth factor 13 (*Fgf13*) mitogen-activated protein kinase 8 interacting protein 3 (*Mapk8ip3*), trafficking protein, kinesin binding 2 (*Trak2*), Jagged 1 (*Jag1*), serine/threonine kinase 3 (*Stk3*), and kruppel-like factor 9 (*Klf9*).

### Semi-quantitative real-time reverse transcriptase polymerase chain reaction

**Figure 2:**
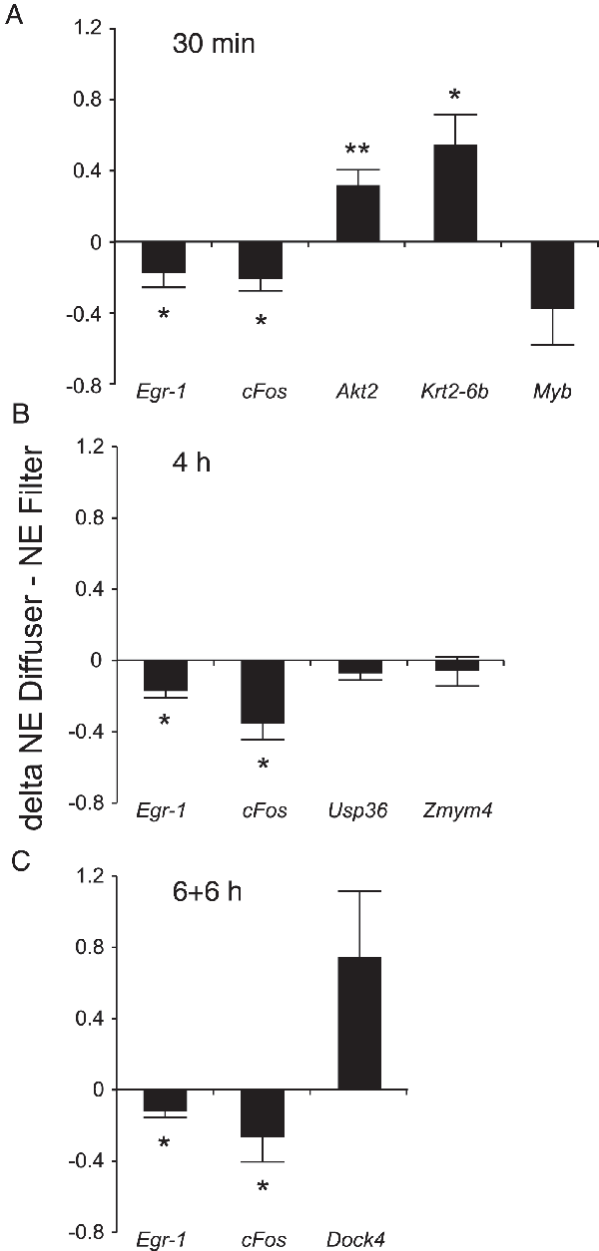
Analysis of GeneChip® results by real-time reverse transcriptase polymerase chain reaction. Delta values were calculated between the normalized expression values of the diffuser- and neutral density (ND) filter-treated eyes and are plotted on the ordinate. Six mice were used for each experiment. Results were given as follows for each treatment group: (**A**) 30 min treatment group, (**B**) presents 4 h group and (**C**) 6+6 h group. Single asterisk represents p<0.05 *; while double asterisk indicates p<0.01 **. Error bars denote standard errors of the mean.

mRNA expression levels were evaluated by real-time RT-PCR. Six animals were used in each group. Genes chosen for validation met certain criteria. First, the signal intensity in either group (diffuser or ND filter) exceeded 65, and second, the Affymetrix probe sets covered a coding region (as determined by the University of California Santa Cruz genome browser). The genes shown in Table 3 were identified after gcRMA normalization of the data set. A previous analysis of the 30 min experiment using MAS5 normalization resulted in a higher number of differentially regulated genes and the confirmation of three genes (*Akt2*, *Krt2-6b*, *Myb*) from that list. The issue of normalization is dealt with in the Discussion section. Accordingly, eight potentially interesting genes were selected for validation: early growth response 1 (*Egr-1*), protein kinase B (*Akt2*), keratin complex 2, basic, gene 6b (*Krt2-6b*), myeloblastosis oncogene (*Myb*), FBJ osteosarcoma oncogene (*cFos*), ubiquitin specific peptidase 36 (*Usp36*), zinc finger, MYM-type 4 (*Zmym4*), and dedicator of cytokinesis 4 (*Dock4*). The mRNA expression levels of these genes were measured in diffuser- and ND filter-treated eyes by RT-PCR (Figure 2).

The differential expression of most genes could be confirmed by semi-quantitative real-time RT-PCR. However, the differential expression of *Dock4* (p=0.07) and *Myb* (p=0.09) just missed significance, and the differential expression of *Usp36* and *Zmym4* also could not be confirmed. The correlation (Table 4) between the results obtained by the GeneChip® experiment and by real-time PCR was good (R=0.86; p<0.01).

**Table 4:** Correlations between results obtained by microarray and real-time polymerase chain reaction analysis.

**Gene**	**Time**	**Microarray**	**PCR**
*Egr-1*	30 min	-1.65	-1.49
*Akt2*	30 min	1.73	1.34
*Krt2-6b*	30 min	14.1	3.08
*Myb*	30 min	-2.22	-1.64
*Egr-1*	4 h	-2.2	-1.97
*Fos*	4 h	-2.05	-2.1
*Usp36*	4 h	1.61	-1.13
*Zmym4*	4 h	-1.95	-1.07
*Egr-1*	6+6 h	-1.77	-2.05
*Dock4*	6+6 h	1.56	1.37

The changes that were obtained by the two methods are expressed as the ratio of the expression level in the diffuser-treated eyes to the expression level in the ND filter-treated eyes.

### Expression of *Egr-1* and *cFos* mRNA as a function of time

**Figure 3:**
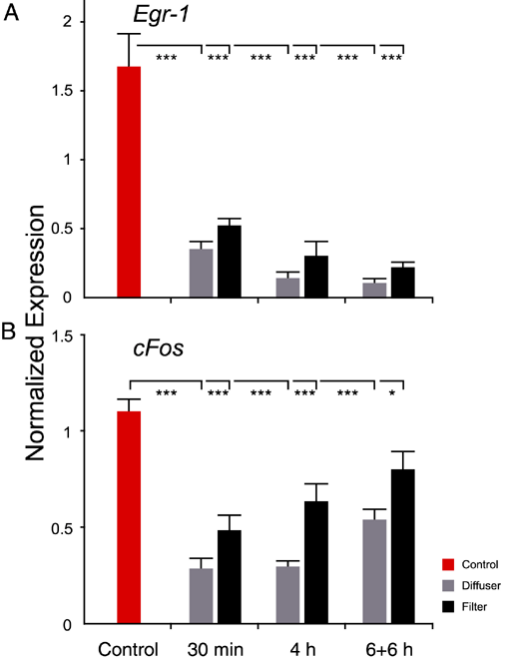
Expression of *Egr-1* and *cFos* in control animals and as a function of time in treated animals. The mRNA expression of *Egr-1* (**A**) and *cFos* (**B**) in diffuser- and neutral density (ND) filter-treated eyes is displayed as a function of time. Normalized expression values were plotted on the ordinate. Error bars denote standard errors of the mean (SEM). Statistical results represent the ANOVA followed by Dunnett's test (comparisons with a control). Single asterisk indicates p<0.05 *; while triple asterisk denotes p<0.001 ***. Three control animals and six treated animals were used for each experiment.

The impact of diffuser and ND filter treatment on *Egr-1* and *cFos* mRNA expression was analyzed over time and also compared to a group of untreated control animals (Figure 3).

The *Egr-1* mRNA level in the eyes of treated animals was remarkably reduced when compared to that in the control group. Real-time RT-PCR data were analyzed statistically by ANOVA followed by Dunnett's test (p<0.001). After 30 min, the average *Egr-1* mRNA level dropped to about 26% of the control group level. After 4 h of treatment the mean mRNA expression level was about 14% of the initial value, and at the end of the 6+6 h treatment, mean mRNA expression level was approximately 10% of the baseline level. The reduction of image intensity therefore had a higher impact on *Egr-1* expression level than did the changes in image contrast and spatial frequency content that were induced by diffuser wear. Nevertheless, the mRNA expression differences (Figure 2) between the diffuser- and ND filter-treated eyes persisted for 6+6 h.

*cFos* mRNA expression was also found to be decreased by the treatment in comparison to the control group. The real-time RT-PCR data were analyzed statistically by ANOVA followed by Dunnett's test (C versus ND Filter 24 h: p<0.05; C versus all other groups: p<0.001). It was found that after 30 min, the average *cFos* mRNA level dropped to 35% of the baseline level but increased subsequently. After 4 h the mean mRNA expression level was approximately 44% of the initial value, and after 6+6 h the mean mRNA expression level increased by 17% reaching 61% of the control group level. As in the case of *Egr-1*, the reduction of image intensity had a high impact on *cFos* expression, but differences between diffuser- and ND filter-treated eyes were still apparent after 6+6 h (Figure 2).

### Baseline expression levels of *Akt2*, *Krt2-6b*, *Myb*, *Zmym4*, *Usp36*, and *Dock4*

**Figure 4:**
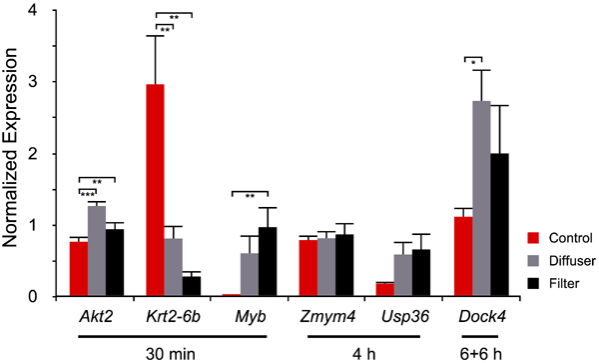
Retinal mRNA expression levels in untreated (control) and treated (diffuser/filter) animals. Normalized expression values were plotted on the ordinate. Error bars denote standard errors of the mean (SEM). Statistical results represent the ANOVA followed by Tukey-Kramer test. A single asterisk indicates p<0.05 *, while a double asterisk denotes p<0.01 **, and a triple asterisk marks p<0.001 ***. Three control animals and six treated animals were used in these experiments.

Baseline expression level of the genes chosen for validation was evaluated by measuring target gene expression in an untreated control group. The control group was sacrificed after 6 h of light exposure to circumvent diurnal influences on mRNA levels (Figure 1A). Real-time RT-PCR data (Figure 4) were analyzed statistically by ANOVA followed by Tukey-Kramer HSD tests.

The mRNA expression of *Akt2* and *Dock4* increased in both diffuser- and ND filter-treated eyes, however, the increase was more pronounced in the diffuser-treated eyes. In contrast, *Krt2-6b* expression was substantially high in untreated animals, while it was lower in the treated animals. *Myb* expression was hardly detectable in the control group and therefore seems to be induced by the treatment conditions. *Usp36* mRNA level seemed to be higher in diffuser- and ND filter-treated eyes, although not statistically significant. *Zmym4* mRNA expression was not influenced by either treatment condition.

### Semi-quantitative real-time reverse transcriptase polymerase chain reaction

**Figure 5:**
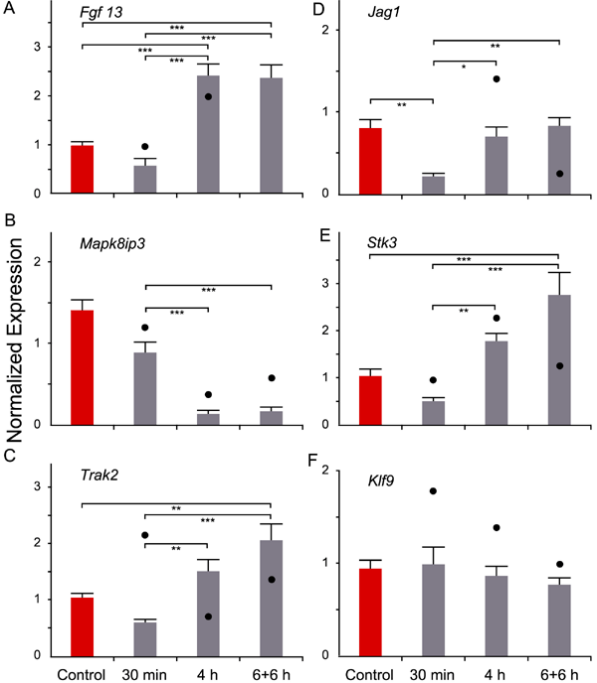
mRNA expression levels in the diffuser-treated eyes as a function of time. Normalized expression values for Fgf13 (**A**), Mapk8ip3 (**B**), Trak2 (**C**), Jag1 (**D**), Stk3 (**E**), and Klf9 (**F**) were plotted on the ordinate. Each black dot represents the approximate expression level changes as determined by the GeneChip® experiment. Error bars denote standard errors of the mean (SEM). Statistical results represent the ANOVA followed by Tukey-Kramer test. A single asterisk indicates p<0.05 *, a double asterisk equals p<0.01 **, and a triple asterisk representsp<0.001 ***. Three control animals and six treated animals were used for each experiment.

Six potentially interesting genes were selected to validate the diffuser specific expression changes over time: *Fgf13*, *Mapk8ip3*, *Trak2*, *Jag1*, *Stk3*, and *Klf9* mRNA. Their levels were measured in diffuser-treated eyes only. Baseline expression levels were determined in untreated control animals that spent 6 h in light, including 30 min in the rotating drum. The statistical analysis was done by ANOVA followed by pair-wise Tukey-Kramer HSD post hoc tests (Figure 5).

The microarray results were fully confirmed by real-time RT-PCR for *Fgf13* (Figure 5A) and *Mapk8ip3* (Figure 5B), in part for *Stk3* (Figure 5E), and *Trak2* (Figure 5C), but not for *Klf9* (Figure 5F), and *Jag1* (Figure 5D). Possible reasons for this are addressed in the Discussion section. For the purpose of clarity, not all significant changes are shown in Figure 5B but were as follows: the *Mapk8ip3* mRNA level was significantly decreased in the treatment groups when compared to the control group (control versus 30 min: p<0.01; control versus 4 h and 6+6 h: p<0.001).

### Network analysis

Molecular networks comprising the differentially regulated genes were generated through the use of Ingenuity Pathways Analysis. The relatively low number of differentially expressed genes could not be consigned to a specific pathway. After 30 min and 4 h of deprivation, most of the genes that changed were involved in cellular development, whereas after 1 day of treatment, they were mostly involved in DNA replication, recombination, nucleic acid metabolism, and small molecule biochemistry.

## Discussion

Placing diffusers over the eyes can artificially induce myopia in animal model. The identification of retinal genes and proteins that are induced or repressed by such conditions can provide new candidates for pharmacological intervention of myopia also in humans. The present study demonstrated that short as well as longer periods of retinal image degradation cause significant changes in the expression level of some genes.

### *Egr-1* mRNA expression

The influence of form deprivation on *Egr-1* mRNA expression [21] could be confirmed and strengthened in the present study, since *Egr-1* expression was decreased in the diffuser-treated eyes at all time points even though the intensity of the retinal images was matched in both eyes. Moreover, Egr-1 expression in the retina was strongly regulated by light. In other species it was shown that in a subset of cells, Egr-1 was not regulated by the light intensity but instead regulated by the sign of defocus (i.e. the glucagonergic amacrine cells in the chicken retina and the GAD65 cells in the macaque retina). In these cells, Egr-1 might induce growth signals independently from the lighting situation. We suggest that there exists a focus-sensitive subpopulation of Egr-1 expressing cells in the murine retina as well.

The early growth response protein 1 (Egr-1) [30] was first identified as an immediate early gene responsive to growth factors and various differentiation signals and later confirmed as a transcriptional regulatory protein. It is induced in the absence of de novo protein synthesis by mitogens, developmental or differentiation cues, tissue or radiation injury or signals that cause neuronal excitation [31]. The zinc-finger protein Egr-1 is located in the nucleus [32-34] and has numerous target genes [35], among which are PDGF-A [36] and PDGF-B [37], bFGF [38] and TGF-β1 [37,39]. A potential connection between Egr-1 and myopia was first described in the chick, where it was found that the expression of ZENK correlates with the sign of defocus imposed by lenses in a subset of amacrine cells (AC), specifically the glucagon AC [20]. In the macaque retina it was shown that focus-sensitive immunoreactivity for Egr-1 is induced in a subpopulation of GABAergic amacrine cells (GAD65-immunoreactive cells) [22].

### *cFos* mRNA expression

The decreased expression of *cFos* mRNA as a result of reduced image quality was determined by microarray analysis after 4 h of treatment. Furthermore, the analysis by real-time RT-PCR also revealed a down-regulation of *cFos* mRNA in the diffuser-treated eyes after 30 min and even after 6+6 h. The level of *cFos* mRNA, in contrast to that of *Egr-1* mRNA, increased slightly over time in both diffuser and in ND filter-treated eyes, implying that the two genes are independently regulated when the image contrast is reduced by diffusers. The independent regulation of those two immediate early genes has been previously shown in the chick retina [20].

cFos is an immediate early gene that belongs to the activator protein (AP)-1 transcription factor family [40-42]. In the retina, Fos-like protein expression has been found to be induced by light in amacrine cells and ganglion cells of dark-adapted rabbits [43]. The activation of *cFos* in response to light onset, and its circadian regulation, have been described in many studies [44-47]. The role of *cFos* in myopia research has been previously investigated in the chick retina, where it was found that switching from diffuse blur to focused vision induced cFos protein expression in an amacrine cell subpopulation [48]. Thus changes in *cFos* gene expression might indicate the activation of retinal interneurons or circuits that mediate the growth responses to well-focused images. Furthermore, the transcriptional activity of c-Jun and cFos can be inhibited by retinoic acid receptors in response to their ligands [49]. Conversely, AP-1 represses the transactivation of retinoid receptors [50,51]. Retinoic acid has been previously implicated in the regulation of eye growth. The synthesis of retinoic acid is regulated in a focus-dependent manner in the choroid and the retina [52-54] and the expression of retinal retinoic acid receptors was found to be increased under form-deprivation conditions [55].

*Akt2* (also called protein kinase B β, PKBβ) is a serine/threonine protein kinase and a downstream effector of the phosphatidylinositol 3-kinase (PI3K). Many physiological effectors and pharmaceuticals, including insulin, insulin-like growth factor, vascular endothelial growth factor, nerve growth factor, carbachol, forskolin and vanadate, are capable of inducing Akt kinase activity, primarily in a PI3K-dependent manner [56]. However, it has been found that Akt is also activated independently of the PI3K by heat shock and hyperosmolarity [57] as well as oxidative stress and chemical stressors [58]. Akt was first implicated in signal transduction by the demonstration that its kinase activity is induced by growth factors such as platelet derived growth factor (PDGF) and basic fibroblast growth factor (bFGF) [59,60]. Furthermore, it has been shown that Akt2 detaches from the inner surface of the plasma membrane, where it is initially activated, and translocates to the nucleus within 30 min of its activation by growth factors [61]. In the nucleus, Akt isoforms have been hypothesized to phosphorylate and modulate the activity of transcription factors [56].

In mammals, Akt2 is expressed in most tissues and organs including the retina, but especially in insulin-responsive tissues [62,63]. Previous experiments of our group have shown that insulin might act as a growth stimulator (unpublished data by MF obtained from studies in the chick). *Akt2* therefore may be a new and interesting candidate for involvement in signal transduction during myopia development.

### *Krt2-6b*, *Myb*, and *Dock4* expression

Fewer details are known about the other genes that were found to be regulated by deprivation: *Krt2-6b* (also called *mK6b*), the expression of which was reduced after 30 min of diffuser treatment and even more after neutral density filter treatment compared to untreated control eyes, encodes an intermediate filament featuring a complex expression pattern. Krt2-6b is constitutively expressed in a variety of internal stratified epithelia but the expression can also be induced by injury and other acute challenges. mRNA is induced in the epidermis as early as 1 h following acute injury or topical application of phorbol esters [64] or retinoic acid [65].

*Myb* (also called *c-myb*) expression was induced by diffuser treatment and to an even higher extent by filter wear. Myb is a myeloblastosis oncogene acting as a transcriptional transactivator. Gene knockout experiments have shown that genes of the Myb family play an essential role in development [66]. Myb is expressed in ganglion, amacrine, horizontal, and photoreceptor cells of adult mice, suggesting that it might play a role in the physiology of retinal cells [67]. Myb family target genes accomplish diverse functions in cell death, cell adhesion, transcription and signal transduction.

*Dock4* is a potent rac activator and an unconventional guanine exchange factor for the Rho family of guanosine triphosphatases (Rho GEF GTPases), as a protein interacting with harmonin, which is expressed in the inner and outer photoreceptor segments. Mutations in the actin bundling and PDZ domain-containing protein harmonin are the causes of Usher syndrome type 1C (USH1C), a syndrome of congenital deafness and progressive blindness [68].

### Data normalization and statistical analysis of microarray experiments

Initially, the data of the 30 min experiment were normalized using MAS5 (Microarray Suite 5.0), resulting in 169 differentially expressed genes (FC 1.5, p <0.01). Since gcRMA normalization is currently recommended as the method of choice by some authors [69], it was subsequently applied to normalize the data of all experiments. Based on this method, the obtained list contained only 16 genes. The same was true for the 4 h and the 6+6 h experiments, where 173 and 164 differentially expressed genes were obtained when MAS5 normalization was conducted, respectively. In a comparison of the MAS5 and gcRMA generated gene lists, only *cFos* was found in both lists obtained for the 4 h experiment. The other lists showed no overlap. This result clearly shows that differences in the normalization method may have a dramatic influence on the outcome of microarray studies. However, no clear consensus exists as to which method is best under a given set of circumstances [70]. A major difference between both normalization strategies is the correction for hybridization to the mismatch (MM) probes for that particular probe set as applied by MAS5 while gcRMA alternatively calculates a background adjustment step that ignores the MM intensities but incorporates sequence information from the probes (GC content) [27].

Although it is obvious that the mode of normalization has a considerable effect, the genes chosen from either list could be confirmed in our case. Both approaches give therefore only an incomplete, and perhaps partially flawed, picture.

It is also important to mention that even though the data were not Benjamini/Hochberg corrected we were nevertheless able to confirm some of the differentially expressed genes which were detected with less rigorous statistical testing. This may suggest that a more pragmatic approach with less statistical rigor, but more repeated experiments, may represent a more successful screening procedure.

The low number of differentially expressed genes might mirror the fact that a limited number of quite specific changes are expected in the retina during eye growth regulation. It seems likely that the circuits that control eye growth have to operate without compromising other retinal and ocular functions, and that they are therefore localized to a sub-set of retinal neurons.

Another reason for the low number of differentially expressed transcripts might also be that, unless a gene is expressed by just one type of cell, the data could conceal opposite changes in expression in two or more cell types. Thus, for example, "zero" difference could result from equal and opposite changes in two cell types, and "increase" could result from a larger increase in one cell type and a lesser decrease in another type of cell.

### Confirmation of microarray results

Most of the changes in gene expression detected by microarray and chosen for further study were validated by RT-PCR. Nevertheless, some of the microarray results, particularly the changes in expression over time, were not confirmed. One reason might be that comparing the independent microarray experiments is more complicated, since the preparations and all following procedures of the different treatment periods were done with some time delay. As previously discussed, normalization is a critical point in microarray analysis, and normalizing the data of all 18 GeneChips® together may carry a risk of potential bias.

Another reason for the difficulties of validation could be that some of the differences between expression in treated and control eyes were minute and therefore difficult to confirm.

### Time kinetics

The identification of genes that are temporally regulated by diffuser treatment would be of interest, even though the confirmation of the results proved to be difficult. We think that the results obtained by real-time RT-PCR are more reliable, considering the issues of normalization.

Unfortunately, there is little information about the genes for which the time kinetics was examined: Longer treatment periods (4 h, 6+6 h diffuser wear) increased *Fgf13* mRNA levels in our study. Its exact function is unknown but *Fgf13* was also found to be up-regulated after light-induced retinal damage [71].

*Mapk8ip3* (also called *Jip3* or JSAP1) mRNA levels were strongly reduced in diffuser-treated eyes. Mapk8ip3 is a scaffold protein and brings together consecutive members of the c-Jun NH_2_-terminal kinase (JNK) signaling cascade [72]. It is expressed exclusively in neurons and binds preferentially to JNK3 and also to the MAPK kinase 7 [73].

### Conclusion and outlook

The microarray technique is a powerful technology, offering the possibility to assay thousands of genes in one reaction. Each method has its limitation, and it will therefore never be possible to identify all gene expression changes using only one method. There is evidence from the literature that the extent of changes in retinal mRNA levels during deprivation is sometimes only small and therefore in the range of the detection limit of the used methods (for example Northern blots or real-time PCR). We set the cut-off level for the discovery of differentially expressed genes to more than 1.5 fold, which also included the risk of missing some differentially expressed genes.

It is important to keep in mind that changes in the mRNA level are not always translated into changes in protein content and that post-translational modifications of proteins may also be involved in eye growth regulation. Therefore, a histological study of Akt2 and cFos protein expression in the mouse retina following deprivation seems to be interesting and is planned. It might also be interesting to investigate the expression of the Jun proteins (c-Jun, JunB and JunD) since they dimerize with Fos family members (cFos, FosB, Fra-1, and Fra-2) to form transcriptionally active complexes [74]. Moreover, the mRNA expression pattern of some candidate genes identified in this screening could also be studied in the chick, which is easier to handle and responds more reliably with development of myopia when diffusers are attached.
